# Low Temperature Synthesis and Characterization of AlScMo_3_O_12_

**DOI:** 10.3390/ma8020700

**Published:** 2015-02-16

**Authors:** Rebecca Truitt, Ilka Hermes, Alyssa Main, Anne Sendecki, Cora Lind

**Affiliations:** Department of Chemistry & Biochemistry, the University of Toledo, Toledo, OH 43606, USA; E-Mails: rebeccatruitt4@gmail.com (R.T.); hermesi@mpip-mainz.mpg.de (I.H.); leright1am@gmail.com (A.M.); annesendecki@gmail.com (A.S.)

**Keywords:** negative thermal expansion, A_2_M_3_O_12_, scandium aluminum molybdate, high resolution diffraction, non-hydrolytic sol-gel chemistry

## Abstract

Recent interest in low and negative thermal expansion materials has led to significant research on compounds that exhibit this property, much of which has targeted the A_2_M_3_O_12_ family (A = trivalent cation, M = Mo, W). The expansion and phase transition behavior in this family can be tuned through the choice of the metals incorporated into the structure. An undesired phase transition to a monoclinic structure with large positive expansion can be suppressed in some solid solutions by substituting the A-site by a mixture of two cations. One such material, AlScMo_3_O_12_, was successfully synthesized using non-hydrolytic sol-gel chemistry. Depending on the reaction conditions, phase separation into Al_2_Mo_3_O_12_ and Sc_2_Mo_3_O_12_ or single-phase AlScMo_3_O_12_ could be obtained. Optimized conditions for the reproducible synthesis of stoichiometric, homogeneous AlScMo_3_O_12_ were established. High resolution synchrotron diffraction experiments were carried out to confirm whether samples were homogeneous and to estimate the Al:Sc ratio through Rietveld refinement and Vegard’s law. Single-phase samples were found to adopt the orthorhombic Sc_2_W_3_O_12_ structure at 100 to 460 K. In contrast to all previously-reported A_2_M_3_O_12_ compositions, AlScMo_3_O_12_ exhibited positive thermal expansion along all unit cell axes instead of contraction along one or two axes, with expansion coefficients (200–460 K) of α_a_ = 1.7 × 10^−6^ K^−1^, α_b_ = 6.2 × 10^−6^ K^−1^, α_c_ = 2.9 × 10^−6^ K^−1^ and α_V_ = 10.8 × 10^−6^ K^−1^, respectively.

## 1. Introduction

**Figure 1:**
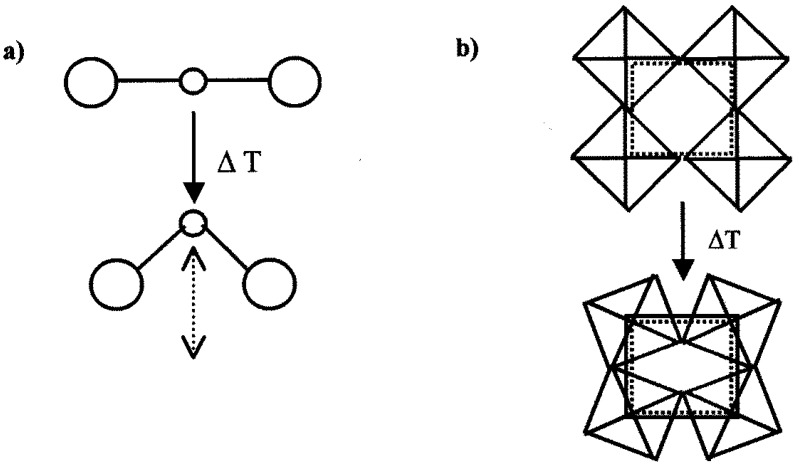
The mechanism of negative thermal expansion due to transverse atomic vibrations: (**a**) transverse motion of an oxygen atom in an M–O–M linkage; and (**b**) cooperative rocking of polyhedra causing a decrease in average metal-metal distances. The squares correspond to the locations of the metal atoms before (solid) and after (dotted) tilting.

Expansion is an important property of a material for many applications [1,2,3,4]. While positive thermal expansion materials are a well-established field, negative thermal expansion (NTE) materials have only been thoroughly studied for a couple of decades [5,6,7,8,9,10,11,12,13,14,15,16,17,18,19,20,21,22,23,24,25,26,27,28,29,30,31,32,33,34,35,36,37,38]. NTE materials contract upon heating, which is described by a negative thermal expansion coefficient α. This unusual behavior is found in a number of corner-sharing polyhedral networks. In most materials, longitudinal vibrations along the bonds between neighboring atoms cause bond expansion upon heating, but other mechanisms outweigh the bond expansion in NTE materials. A frequently found mechanism is the occurrence of low energy phonon modes that induce the tilting of rigid or quasi-rigid polyhedra in corner-shared networks [24,27,39,40,41]. NTE materials that possess stiff M–O bonds and linear M–O–M linkages can exhibit transverse vibrations of the oxygen atoms that decrease the distances between the metal atoms. Such motions can cause coupled rotations of the corner-sharing polyhedra, resulting in contraction of the entire structure (Figure 1).

NTE materials are of particular interest due to their potential to control the expansion of other materials in composites, especially in applications where compounds are subjected to repeated changes in temperature. Ideally, NTE compounds can be integrated as fillers into other materials to form a composite with an altered expansion coefficient, which can be controlled in positive ranges, negative ranges or even held at zero [3,4,42,43,44,45,46,47,48]. An alternative approach that has received recent attention is the formation of substituted NTE materials, in which the intrinsic expansion coefficient of the material can be tuned to match other materials. However, potential applications of these materials in optical or electronic composites, or as substrates or coatings, depend on the absence of discontinuous phase transitions in the working temperature range.

The A_2_M_3_O_12_ family of negative thermal expansion materials, where A is a trivalent metal and M is either molybdenum or tungsten, is of particular interest. A wide range of cations can be substituted into the structure, which can result in materials with a single cation on each site or with cation mixtures on the A or M sites [32,35,49,50]. Many A_2_M_3_O_12_ compounds have been found to display a reversible temperature-induced phase transition between orthorhombic and monoclinic phases at high and low temperatures, respectively. The absence or occurrence of phase transitions depends strongly on the cations present in the structure [35,51]. The monoclinic phase exhibits positive thermal expansion (PTE) for all compositions, while most orthorhombic materials display volume NTE. The expansion behavior in the orthorhombic phase is anisotropic, with NTE along one or two unit cell axes and PTE along the remaining axes. Generally, for compositions that contain a single A-site cation, a lowering of the phase transition temperature is observed for larger or more electropositive cations [51]. Larger cations are also associated with more negative expansion coefficients [52]. In a number of systems, mixing of two trivalent A-site cations results in intermediate expansion coefficients that approximately follow the rule of mixtures and allows the preparation of solid solutions with close to zero thermal expansion [53,54,55,56,57]. However, mixed A-site cations can also result in more complex behavior. For example, Ari *et al.* reported that the phase transition temperatures of Al_2−x_Cr_x_Mo_3_O_12_, Al_2−x_Fe_x_Mo_3_O_12_ and Cr_2−x_Fe_x_Mo_3_O_12_ obey the rule of mixtures, while the expansion coefficients show very distinct behavior in the different systems [58]. For Al_2−x_Cr_x_Mo_3_O_12_ and Cr_2−x_Fe_x_Mo_3_O_12_, a linear decrease in the expansion coefficient with increasing fraction of Cr^3+^ is observed, while Al_2−x_Fe_x_Mo_3_O_12_ compositions show more positive expansion coefficients than either Al_2_Mo_3_O_12_ or Fe_2_Mo_3_O_12_, indicating that other factors must play a role as well. Another interesting observation is that AlInW_3_O_12_ adopts an orthorhombic structure and shows approximately zero volume expansion between room temperature and 1000 K [35]. This is intriguing, as In_2_W_3_O_12_ adopts the monoclinic structure up to 473 K [59], while Al_2_W_3_O_12_ undergoes the monoclinic-to-orthorhombic transition at 267 K [51]. The ability to not only tune expansion coefficients, but also suppress the undesirable phase transition to the monoclinic PTE phase is useful for potential applications [58]. A better understanding of how cation mixing influences phase transition behavior and stabilizes the orthorhombic NTE structure at lower temperatures is clearly desirable.

Low temperature synthetic approaches are attractive for the preparation of mixed metal oxides, as high temperature routes often require large numbers of grind/heat cycles to achieve homogeneous particles. One such method is the non-hydrolytic sol gel (NHSG) process, which was shown to provide an elegant route to mixed metal oxides [60,61,62,63,64]. It was previously demonstrated that this chemistry can be applied to the synthesis of A_2_M_3_O_12_ compounds. Reports included the preparation of Ga_2_Mo_3_O_12_ [65], which cannot be obtained by solid-state routes, a new polymorph of Y_2_Mo_3_O_12_ [66] and the crystallization of MgA^IV^M_3_O_12_ (A^IV^ = Zr, Hf; M = Mo, W) compositions [67]. Many other compositions were obtained at considerably lower temperatures than samples prepared by other methods [68]. This demonstrates that the NHSG approach offers the potential to obtain compounds with cations that do not readily form A_2_M_3_O_12_ compositions (e.g., Ga) and that the intimate mixing in solution can result in more facile crystallization. These observations suggest that this method should also be suitable for combining cations with considerably different ionic radii under optimized reaction conditions. In this paper, we demonstrate the extension of NHSG methods to AlScMo_3_O_12_, a quaternary AA’M_3_O_12_ compound, where A and A’ are two different trivalent metals with very different radii (Sc r_6_^3+^ = 75 pm; Al r_6_^3+^ = 39 pm) [69]. While AlScMo_3_O_12_ can be obtained by traditional ceramic methods [55], the ability to prepare NTE materials at lower temperatures by NHSG chemistry gives access to smaller particles, which are expected to result in better dispersion and property transfer in filler/matrix composites.

Solution-based routes offer much better kinetics than high-temperature, solid-state approaches; however, they may produce inhomogeneous products if different precursors react at different rates in solution. The relative reaction rates can often be modified through the careful choice of reaction variables. Low temperature routes give access to a considerable number of tunable variables, like solvent system, precursor reactivity, concentrations, temperature and time, which allows optimization of the reaction conditions to obtain homogeneous, stoichiometric products. Here, we present a study on the synthesis of AlScMo_3_O_12_ as a function of synthetic variables in NHSG routes. Conditions were optimized to obtain a single-phase homogeneous material with the desired stoichiometry. Samples were characterized by high-resolution diffraction to address whether single-phase powders were obtained, and the expansion behavior was determined by variable temperature diffraction experiments.

## 2. Experimental Section

Reactions were carried out in dried glassware using a glove box and standard Schlenk techniques. Starting metal halides included aluminum chloride (AlCl_3_, Strem Chemicals, 99.99%, Newburyport, MA, USA), scandium chloride (ScCl_3_, Strem Chemicals, 99.99%, Newburyport, MA, USA) and molybdenum chloride (MoCl_5_, Strem Chemicals, 99.6%, Newburyport, MA, USA). In a typical reaction, 0.133 g of AlCl_3_, 0.151 g of ScCl_3_ and 0.820 g of MoCl_5_ were added to a glass ampoule (1:1:3 millimolar ratio). Under stirring, 10 mL of acetonitrile were added. The ampoule was capped with a septum and transferred to a Schlenk line, where 1.8 mL (12.8 mmol) of diisopropyl ether were added by syringe. The solution was allowed to stir for about 1 h during and between the addition of solvent and ether. The ampoule was cooled in liquid nitrogen, evacuated and flame sealed. It was heated at 150 °C for a period of 7 days. The ampoule was then opened in air, and a dark brown/black tar-like product was recovered by evaporation. To obtain a powder, the tar-like product was heated at 300 °C for 3 h. This dark grey powder, which still contains organic residues and is generally amorphous, will be referred to as the “raw” product for the remainder of this paper. The reaction variables explored during synthesis included the type of solvent (CH_3_CN and CHCl_3_), the volume of solvent (3 to 20 mL), temperature (110 to 150 °C) and heating time (3 to 27 days).

Heat treatments were performed on the raw samples to induce crystallization. Raw samples were heated either stepwise to 500, 600 and 700 °C or directly to 700 °C over a period of 2 to 2.5 h, annealed at each temperature for 3 h and quenched in air. Samples were characterized using powder X-ray diffraction (PXRD), thermogravimetric analysis (TGA), scanning electron microscopy (SEM) and energy dispersive X-ray spectroscopy (EDS). PXRD data were collected on a PANalytical X’Pert Pro diffractometer with an X’Celerator detector in Bragg-Brentano geometry using Cu K_α_ radiation. High-resolution PXRD data were obtained at Argonne National Laboratory’s beamline 11-BM at wavelengths of 0.41348, 0.41334 and 0.41388 Å during separate visits. Samples were packed inside 0.8-mm Kapton capillaries. PXRD patterns were analyzed using Jade and the PDF2 database, and refinements were carried out in Topas Academic. Variable temperature diffraction data were also collected at 11-BM at temperatures between 100 and 460 K with approximately 40 K steps between datasets. Thermogravimetric analysis was carried out on a TA Instruments SDT 2960 Simultaneous TGA-DTA to determine the amount of residual organics in the raw samples and the decomposition temperature of the crystalline samples obtained after heat treatment. Air was used as the carrier gas at a flow rate of 110 to 120 mL/min. Raw samples were heated at 10 °C/min to 700 °C, followed by an isothermal treatment for 15 min and cooling to room temperature. A second heating cycle at 10 °C/min to 1200 °C was initiated upon completion of the first cycle. Scanning electron microscopy was carried out on a JEOL JSM-6100 microscope with a Bruker Quantax EDS system with an XFlash 5100 detector. Images were collected at 2 kV on uncoated samples. EDS measurements were carried out at 15 kV using the point, multipoint and mapping approaches. Mapping results were utilized to get a general idea of the sample homogeneity, while numerical results reported in this manuscript were obtained by averaging point or multipoint analysis results for a number of individual grains. Homogeneous samples are expected to give very similar numbers for all point measurements, leading to small statistical errors on the average stoichiometry, while inhomogeneous samples will return different ratios for different grains and the resulting large errors on averages.

## 3. Results and Discussion 

### 3.1. Reaction Completeness and Crystallization Behavior 

All raw samples recovered in this project contained significant amounts of organics, as expected from their dark grey color. TGA data showed between 15 and 80 wt.% of organic residues, which burned off at temperatures up to 500 °C, suggesting that heat treatments should be carried out at this temperature or higher. The second TG-DTA run showed that the crystalline samples decomposed at temperatures around 900 °C. This decomposition is a result of the volatility of MoO_3_. Pure MoO_3_ evaporates above 750 °C, and many molybdates start losing MoO_3_ at similar or slightly higher temperatures. Al_2_Mo_3_O_12_ is known to decompose around 900 °C in TGA experiments, while Sc_2_Mo_3_O_12_ is stable to temperatures in excess of 1200 °C [68]. The decomposition temperature of the samples is similar to Al_2_Mo_3_O_12_, indicating that AlScMo_3_O_12_ may phase separate into Al_2_Mo_3_O_12_ and Sc_2_Mo_3_O_12_ before it decomposes. For a small number of samples, evaporation of 1–5 wt.% of unreacted MoO_3_ was observed at 750 °C, indicating an incomplete reaction. Most samples showed no losses up to 900 °C and total weight losses consistent with the targeted AlScMo_3_O_12_ stoichiometry.

**Figure 2:**
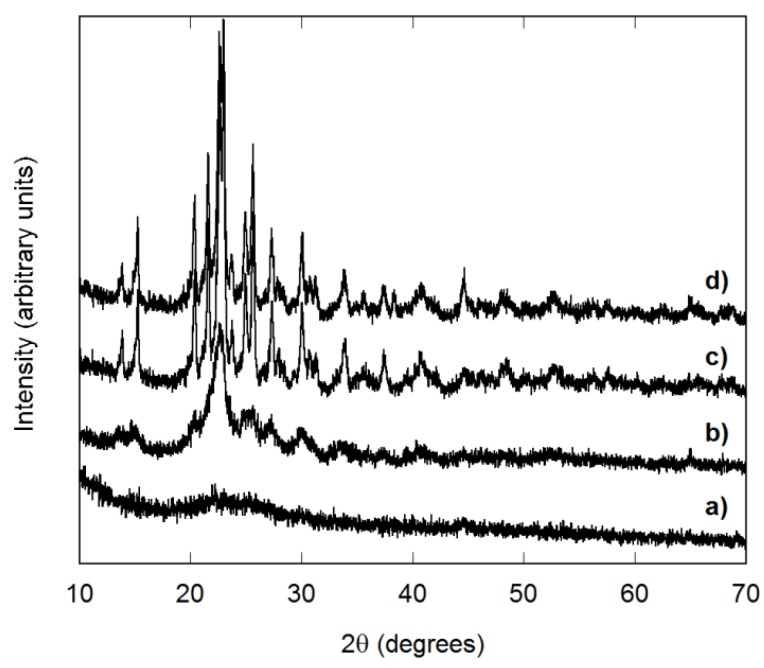
Laboratory PXRD data for AlScMo_3_O_12_ samples: (**a**) as recovered; (**b**) heated to 500 °C; (**c**) heated stepwise to 500, 600 and 700 °C; and (**d**) heated directly to 700 °C.

All raw samples recovered in this project were amorphous (Figure 2a). PXRD studies showed starting crystallization after heating to 500 °C for 3 h (Figure 2b), but samples recovered after 500 or 600 °C heat treatments were still not fully crystalline. Continued heating to 700 °C for 3 h generally resulted in well-crystallized samples (Figure 2c). Most samples matched the PDF card for Fe_2_Mo_3_O_12_ (33-0661), which suggests approximately stoichiometric AlScMo_3_O_12_, as the size of the Fe^3+^ cation in octahedral coordination is halfway between Al^3+^ and Sc^3+^. As direct heat treatments of raw samples to 700 °C gave equal or better crystallinity (Figure 2d), most samples were directly heated to 700 °C for 3 h. Longer heating at 700 °C (up to 24 h) did not improve crystallinity, and heating at 750 °C or higher led to weight loss due to MoO_3_ evaporation and a shift in peak positions towards those of the PDF card for Sc_2_Mo_3_O_12_ (72-2078). The observation of significant weight losses during extended heat treatments (12 wt.% after 24 h at 800 °C) indicates that the higher decomposition temperatures observed by TGA are a result of the fast heating rate, which does not allow the sample to reach thermal equilibrium.

### 3.2. Structure and Homogeneity of Samples 

Al_2_Mo_3_O_12_ and Sc_2_Mo_3_O_12_ both belong to the A_2_M_3_O_12_ family; however, they adopt different structures at room temperature, with Al_2_Mo_3_O_12_ displaying the monoclinic A_2_M_3_O_12_ structure below 473 K [70], while Sc_2_Mo_3_O_12_ crystallizes in the orthorhombic A_2_M_3_O_12_ phase above 178 K [71]. This made it necessary to investigate whether AlScMo_3_O_12_ forms the monoclinic or orthorhombic A_2_M_3_O_12_ structure at room temperature. These structures are closely related, and it is also possible that samples consist of multiple phases with different compositions. Laboratory PXRD data can be used to identify whether samples form two distinct Al- and Sc-rich phases, which result in two overlapping patterns, but are not adequate to address more subtle differences. Samples may be composed of several phases with similar compositions or of particles with a continuous variation in stoichiometry, which results in broad, unresolved peaks in laboratory data. High resolution data can resolve small differences in lattice constants and may reveal split peaks corresponding to multiple phases in cases when laboratory data give single, broad peaks. 

All samples prepared in this research were characterized by high resolution diffraction experiments at a synchrotron. Initially, data were visually inspected to determine whether samples were composed of a single phase or whether multiple phases were present. Samples with broad, completely unresolved peaks in the synchrotron data were not further analyzed, while all other datasets were subjected to refinements to extract lattice constants and structural information. It was found that single-phase, stoichiometric AlScMo_3_O_12_ samples crystallized in the orthorhombic A_2_M_3_O_12_ structure in space group Pnca. All single-phase samples were successfully analyzed by Rietveld refinement, while multiphase samples were either refined by the Rietveld method or treated by the Pawley method when too many phases were present for stable Rietveld refinements. 

**Figure 3:**
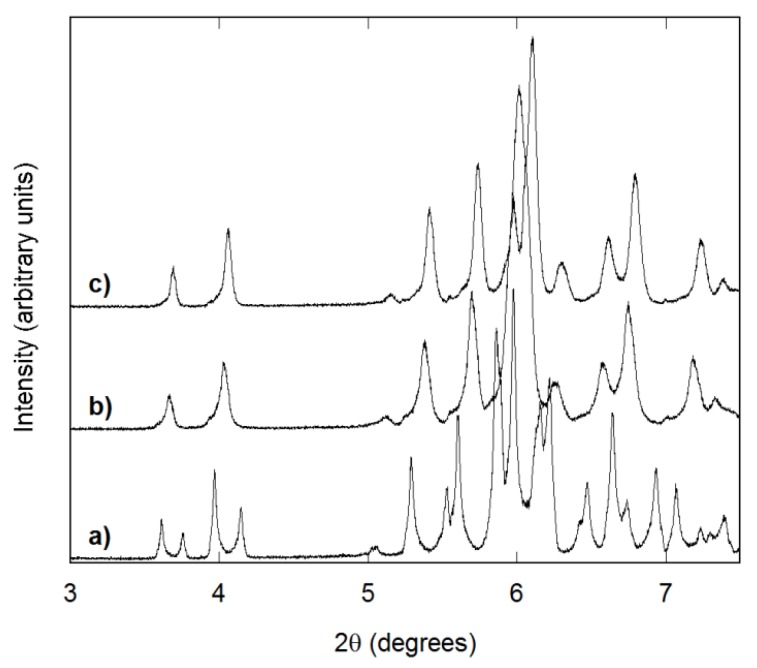
Synchrotron diffraction patterns of AlScMo_3_O_12_ samples prepared in 15 mL of CH_3_CN by heating at 130 °C for: (**a**) 7 days; (**b**) 17 days; and (**c**) 27 days.

Most samples prepared in CH_3_CN showed split or broad peaks, even in laboratory PXRD patterns, which indicates inhomogeneous samples with several distinct compositions or a wide range of similar compositions, respectively. Temperature and reaction time affected the crystallinity of the samples. Reactions at 130 °C required long heating times (3–4 weeks) to obtain single-phase material, and most samples still showed a shoulder corresponding to a small fraction of a scandium-rich phase (~10%–15%) in the high resolution data. Shorter times resulted either in split or very broad peaks (Figure 3). A solvent volume of 10 or 15 mL gave the sharpest peaks. The change in homogeneity with time suggests that the Al_x_Sc_2−x_Mo_3_O_12_ compositions have some solubility in CH_3_CN and that the quaternary compound AlScMo_3_O_12_ is thermodynamically favored, most likely due to entropic contributions.

Reactions in CH_3_CN at 150 °C produced samples with broad or split peaks after three to seven days. Reasonable crystallinity was obtained after two weeks; but longer heating (up to four weeks) did not improve homogeneity, and the peaks always remained broad. Several reactions at 170 °C were also conducted with varying solvent volumes. Most samples showed broad peaks, while a few samples resulted in sharp, single peaks. No trends as a function of solvent volume were obvious, and samples reproduced under identical conditions could give sharp or broad peaks, indicating that other factors, like the presence of nucleation sites on the glassware, may have influenced the outcome. Reactions at 110 °C were not attempted in CH_3_CN, as unreasonable reaction times would be necessary to produce homogeneous samples. TGA experiments on raw samples showed weight losses ranging from 20 to 50 wt.% for 130 °C reactions, 35 to 60 wt.% for 150 °C reactions and 50 to 70 wt.% for 170 °C reactions, indicating that higher reaction temperatures resulted in the incorporation of larger quantities of organic residues in the precipitates. The presence of large quantities of organic material may also contribute to the poor reproducibility of reaction outcomes at 170 °C.

**Figure 4:**
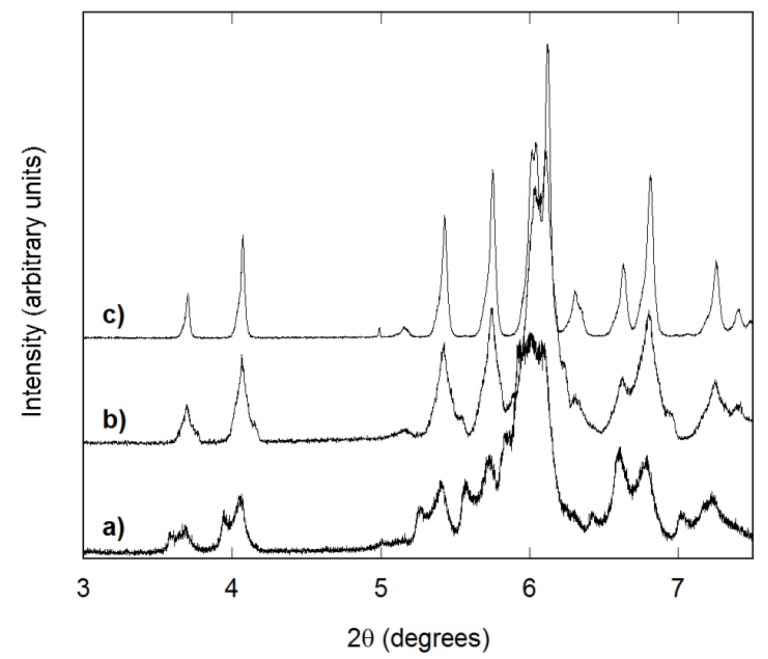
Synchrotron diffraction patterns of AlScMo_3_O_12_ samples prepared in 10 mL of CHCl_3_ by heating at 110 °C for: (**a**) 3 days; (**b**) 7 days; and (**c**) 12 days. All samples were crystallized at 700 °C for 3 h.

Reactions carried out in CHCl_3_ showed a strong dependence on reaction temperature. All samples prepared at 150 °C gave X-ray patterns with clearly split peaks, indicating the formation of several A_2_M_3_O_12_ phases with different compositions. Reactions at 130 °C generally resulted in broad peaks in the laboratory PXRD patterns, especially for large (20 mL) and small (5 mL) solvent volumes. It was found that decreasing the reaction temperature to 110 °C gave better homogeneity. In addition, increasing the reaction time resulted in sharper peaks, with 3-day reactions producing broad, split peaks, while gradual sharpening was observed after seven days of reaction and sharp peaks for 12-day reactions (Figure 4). As the results for reactions at 110 to 150 °C clearly indicated that lower temperatures gave superior homogeneity, reactions at 170 °C were not carried out in CHCl_3_. TGA analysis showed that the amount of organic residues increased with reaction temperature (15–45 wt.% at 110 °C, 40–70 wt.% at 130 °C and 55–80 wt.% at 150 °C), similar to reactions in CH_3_CN. Overall, the amount of residual organics was slightly higher for reactions in CHCl_3_. This could be related to the poorer solubility of many metal halides in this solvent compared to CH_3_CN.

While the observation of unsplit, sharp peaks is an indicator of single-phase, homogeneous samples, it is also important to address whether the target composition AlScMo_3_O_12_ was achieved. As a first approximation, Al_x_Sc_2−x_Mo_3_O_12_ can be regarded as a solid solution of Al_2_Mo_3_O_12_ and Sc_2_Mo_3_O_12_, and the composition of samples can be estimated from PXRD data through extraction of lattice constants using Vegard’s law. However, it is important to keep in mind that Al_2_Mo_3_O_12_ adopts the monoclinic A_2_M_3_O_12_ structure below 473 K [70], while Sc_2_Mo_3_O_12_ crystallizes in the orthorhombic A_2_M_3_O_12_ phase above 178 K [71]. The monoclinic cell is a distorted superstructure of the orthorhombic phase, with a cell volume twice that of the high-temperature structure [71]. The phase transition from monoclinic to orthorhombic symmetry usually involves a discontinuous change in cell volume per formula unit of about 1.5%–2%. Thus, Vegard’s law calculations could be set up in two different ways, by using half the cell volume of monoclinic Al_2_Mo_3_O_12_ as the 100% Al boundary or by extrapolating the cell volume of orthorhombic Al_2_Mo_3_O_12_ at room temperature. This extrapolation is not trivial, as the volume expansion of orthorhombic Al_2_Mo_3_O_12_ is not linear, but instead changes from small positive up to 775 K to negative at higher temperatures [70]. For this reason, calculations were set up using the monoclinic Al_2_Mo_3_O_12_ cell volume. Analyses were carried out on high-resolution synchrotron data, which facilitated the assignment of samples as single-phase or multiple phases. For a few samples, data were not refinable, as the patterns consisted of too many overlapping peaks. For all other samples, single or multiphase Rietveld refinements were set up in Topas Academic. It was found that the cell volumes of most single-phase samples corresponded to compositions between Al_1.2_Sc_0.8_Mo_3_O_12_ and Al_0.8_Sc_1.2_Mo_3_O_12_. Similar results were obtained for the major phase in samples with one major and one smaller secondary phase. Refinement results for selected samples are summarized in Table 1. 

**Table 1:** Reaction conditions and refinement results for selected AlScMo_3_O_12_ samples.

T_synth_ (°C)	Solvent	V_solv_ (mL)	t_synth_ (days)	# of phases	Space group	Phase fraction	V (Å^3^)	%Sc Vegard
130	CH_3_CN	10	7	4+	Pnca *	11%	1022.11	4
						31%	1088.09	36
						27%	1123.28	54
						31%	1153.42	68
130	CH_3_CN	10	17	2	Pnca	89%	1124.95	54
					Pnca	11%	1184.49	84
130	CH_3_CN	10	27	2	Pnca	86%	1107.58	46
					Pnca	14%	1154.62	69
150	CH_3_CN	9	14	2	Pnca	74%	1125.15	54
					Pnca	26%	1173.71	78
170	CH_3_CN	9	7	1	Pnca	100%	1115.99	50
170	CH_3_CN	9	7	2	Pnca	56%	1075.86	30
					Pnca	44%	1131.80	58
110	CHCl_3_	5	3	2	Pnca	71%	1112.73	48
					Pnca	29%	1200.11	91
110	CHCl_3_	10	7	1	Pnca	100%	1100.98	43
110	CHCl_3_	15	12	1	Pnca	100%	1102.11	43
130	CHCl_3_	15	7	2	P 2_1_/c	6%	2060.65	8
					Pnca	94%	1121.07	52

* All phases were modeled as orthorhombic to ensure stable refinement.

**Figure 5:**
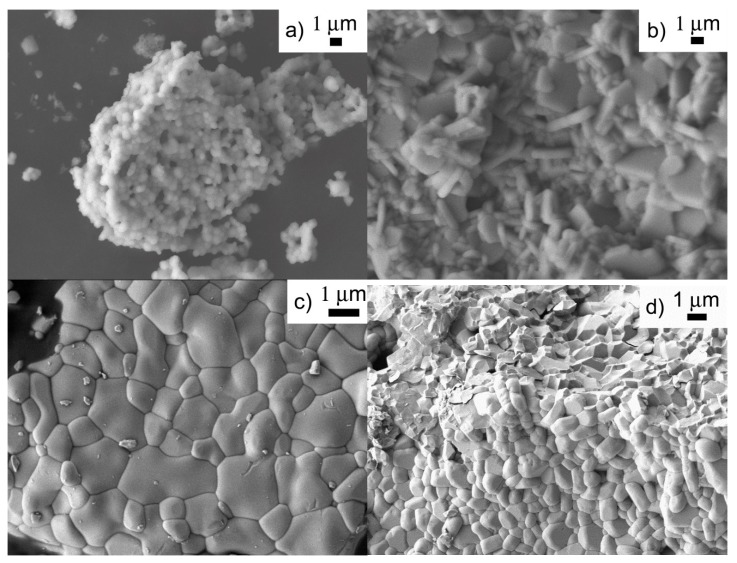
SEM images of samples prepared in: (**a**) CH_3_CN at 150 °C; (**b**) CH_3_CN at 170 °C for 7 days; (**c**) CHCl_3_ at 170 °C; and (**d**) CHCl_3_ at 110 °C. All images were recorded using heat-treated samples.

SEM images of the crystalline samples showed particle sizes ranging from 100 nm to 2 µm. Most samples prepared in CH_3_CN were composed of small, rounded particles that formed agglomerates (Figure 5a). Only a couple of acetonitrile samples showed distinctly faceted particles (Figure 5b). In contrast, all samples recovered from reactions in CHCl_3_ consisted of dense conglomerates of impinging grains (Figure 5c,d). The particle sizes of chloroform samples were clearly limited by neighboring grains impeding further growth. EDS spot analysis confirmed Al:Sc:Mo compositions ranging from (1.1 ± 0.2):(0.9 ± 0.3):3 to (0.9 ± 0.2):(1.3 ± 0.4):3 (all measurements were normalized to the amount of Mo) for single-phase samples with unit cell volumes corresponding to 40%–60% Al and Sc occupancy. In contrast to the results for single-phase samples, averaging of EDS spot analysis results for samples with multiple A_2_M_3_O_12_ phases resulted in very high standard deviations, indicating that samples contained a range of compositions in different grains.

### 3.3. Expansion Behavior of AlScMo_3_O_12_

The intrinsic expansion behavior of AlScMo_3_O_12_ was determined by variable temperature X-ray diffraction experiments at 11-BM, using a highly crystalline and stoichiometric sample (prepared in CHCl_3_ at 110 °C for 12 days). Data were collected at temperatures between 100 and 460 K, and lattice constants were extracted by Rietveld refinement (Table 2). All unit cell axes showed positive expansion. This result was surprising, as A_2_M_3_O_12_ compounds that adopt the Pnca polymorph generally display NTE along one or two unit cell axes, with positive expansion along the remaining axes, which can result in positive, zero or negative volume expansion. Our observations also differ from the results obtained by Wu *et al.* on Al_x_Sc_2−x_Mo_3_O_12_ samples prepared by ceramic methods [55], who reported positive α_b_ and α_c_ values for compositions with at least 50% Al, paired with a small negative α_a_ value for AlScMo_3_O_12_, and approximately zero expansion along the a-axis for Al_0.7_Sc_1.3_Mo_3_O_12_. This could be due to the different temperature ranges investigated (100 to 460 K *versus* 298 to 1073 K) or result from small errors in lattice constants due to changes in sample height that commonly occur in laboratory variable temperature XRD experiments. Such changes are difficult to correct unless data are collected over a wide angular range on samples mixed with an internal standard. In addition, the samples measured by Wu *et al.* were prepared by firing at 800 °C for 24 h. In our hands, such heat treatments resulted in significant weight losses (~12%) due to evaporation of MoO_3_ for both NHSG and solid-state samples. Thus, it is possible that the starting stoichiometry was not preserved in the previous study, as no elemental analysis was presented. It is interesting to note here that even after complete decomposition at 1100 °C, the strong, sharp Sc_2_Mo_3_O_12_ peaks made it impossible to detect the Al_2_O_3_ phase, which must also be present. Thus, partial decomposition of a sample may not be detectable by XRD methods. A further complication in the analysis of Al_x_Sc_2−x_Mo_3_O_12_ prepared by ceramic methods may result from the broad peaks observed for compositions containing 50% to 85% Al on the A-site. This could indicate slightly inhomogeneous samples.

**Table 2:** Rietveld refinement results of variable temperature diffraction data of AlScMo_3_O_12_.

T (K)	R_p_ (%)	a (Å)	b (Å)	c (Å)	β (°)	V (Å^3^)	%Sc
100	11.7	15.771	9.291	18.382	125.64	2189.04	46.4
100	9.2	9.292	12.816	9.191	90.0	1094.54	51.6
140	9.1	9.299	12.820	9.201	90.0	1096.91	50.9
180	8.7	9.307	12.825	9.211	90.0	1099.40	50.7
220	8.4	9.309	12.828	9.215	90.0	1100.55	50.6
260	8.6	9.311	12.832	9.218	90.0	1101.26	50.5
295	8.5	9.312	12.835	9.219	90.0	1101.85	50.4
320	8.7	9.312	12.836	9.220	90.0	1102.04	50.6
340	8.8	9.312	12.838	9.220	90.0	1102.22	50.7
380	9.0	9.313	12.841	9.221	90.0	1102.70	50.5
420	9.0	9.313	12.845	9.222	90.0	1103.16	52.0
460	9.1	9.313	12.847	9.222	90.0	1103.47	50.4

**Figure 6:**
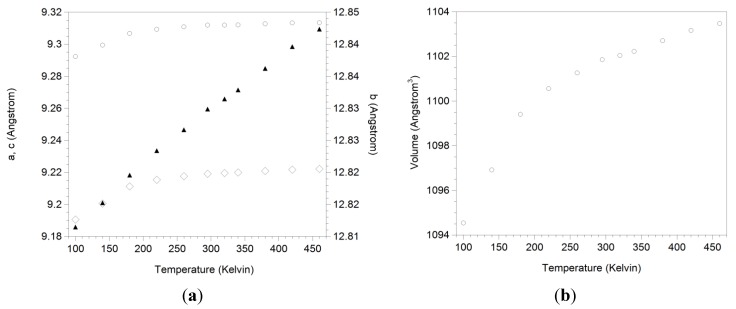
Expansion data of (a) unit cell axes a (◇), b (▲) and c (○); and (b) unit cell volume of AlScMo_3_O_12_.

Our results make AlScMo_3_O_12_ the first reported material crystallizing in the orthorhombic Sc_2_W_3_O_12_ structure that does not show zero expansion or NTE along any cell axis. The expansion behavior of all axes was linear above 200 K. The relative expansion coefficients for the 200 to 460 K temperature range were determined to be α_a_ = (1.7 ± 0.2) × 10^−6^ K^−1^, α_b_ = (6.2 ± 0.1) × 10^−6^ K^−1^, α_c_ = (2.9 ± 0.3) × 10^−6^ K^−1^ and α_V_ = (10.8 ± 0.6) × 10^−6^ K^−1^, respectively. A significant contraction of the a- and c-axes was observed upon cooling below 200 K (Figure 6). These data were excluded from the calculation of expansion coefficients. The more pronounced decrease in cell volume below 200 K suggested that the material may be approaching the orthorhombic to monoclinic phase transition. To address whether the monoclinic phase had already formed at 100 K, this dataset was refined using both the orthorhombic and monoclinic structures. The orthorhombic structure still provided a better fit to the data, indicating that the phase transition must occur below 100 K. Unfortunately, the available experimental setup does not allow collection of data at lower temperatures. DSC did not show any evidence of a phase transition above 110 K either.

Our results indicate that the mixing of Al and Sc on the A-site resulted in a significant suppression of the orthorhombic to monoclinic phase transition temperature to a temperature at least 80 K lower than that of either parent compound. This is a significant observation and, to our knowledge, constitutes the first time that such a significant suppression of the phase transition has been characterized by high-resolution diffraction studies combined with Rietveld analysis, which can distinguish the small structural differences between the orthorhombic and monoclinic polymorphs much more reliably than any other method. Generally, phase transition temperatures between those of the parent compounds are observed, which in many cases follow Vegard’s law. The only well-established exception to this is AlInW_3_O_12_, which is expected to undergo the phase transition below room temperature [35]. Unfortunately, the exact transition temperature has never been reported. The only other report of a significantly lower phase transition temperature is found in Li’s study of Fe_2−x_Y_x_Mo_3_O_12_ [72], which stated that Fe_1.5_Y_0.5_Mo_3_O_12_ remained orthorhombic to temperatures below 103 K. This conclusion was based on the absence of significant changes in Raman spectra down to 103 K. However, the changes in Raman spectra are fairly subtle and should be backed up by other methods. The assignment of an orthorhombic structure was based on visual interpretation of laboratory XRD patterns. Close inspection of the patterns displayed in the paper shows the presence of a small peak at the same angle where a characteristic peak for the monoclinic phase would be expected. Thus, the absence of a phase transition upon cooling could also be due to the fact that the material is already monoclinic at room temperature. It would be interesting to study Fe_2−x_Y_x_Mo_3_O_12_ compositions by variable temperature high-resolution diffraction and Rietveld analysis to conclusively establish their structures. 

The exact cause of the unprecedented suppression of the phase transition temperature requires further investigation. It is possible that the significant difference in size of the trivalent cations (${\text{r}}_{\text{A}{\text{l}}^{3+}}/{\text{r}}_{\text{S}{\text{c}}^{3+}}=0.72$) contributes to this behavior. This would also suggest a significant lowering of the phase transition temperature for other cation combinations that result in similar differences. It is interesting to note that the difference in ionic radii between aluminum and indium is even more pronounced (In r_6_^3+^ = 80 pm) and that AlInW_3_O_12_ does not transform to an orthorhombic structure above room temperature. A low-temperature study aimed at determining its phase transition temperature, as well as an investigation of AlInMo_3_O_12_ could establish whether the size difference of the A-site cations is the determining factor in the lowering of the transition temperature. The preparation and study of mixed cation systems containing Al^3+^ and Y^3+^ or a small lanthanide could further elucidate whether our hypothesis is correct.

## 4. Conclusions

The synthesis of AlScMo_3_O_12_ was optimized using the NHSG method at low temperatures. Syntheses should be carried out in CHCl_3_ at low temperatures for extended periods of time. The most homogeneous Al_x_Sc_2−x_Mo_3_O_12_ samples with a 1:1:3 molar ratio of Al:Sc:Mo were obtained at 110 °C in 10 mL of CHCl_3_ with reaction times of 12 days. Heat treatments of raw samples at 700 °C for as short as 3 h produced highly crystalline products. Longer heat treatments at 700 °C did not improve crystallinity, indicating that crystallinity was not due to sintering, but rather due to atomic-level homogeneity in the raw samples. All single-phase samples adopted the orthorhombic A_2_M_3_O_12_ structure, and this phase persisted at temperatures down to 100 K. It seems likely that the material approaches the orthorhombic to monoclinic phase transition at this temperature based on the evolution of unit cell volume with temperature. This temperature is considerably lower than the phase transition temperature of either Al_2_Mo_3_O_12_ or Sc_2_Mo_3_O_12_. In contrast to all other materials in the A_2_M_3_O_12_ family for which expansion data have been reported, AlScMo_3_O_12_ displays positive thermal expansion along all unit cell axes.
